# The Relationship between Insufficient Sleep and Self-Rated Health in a Nationally Representative Sample

**DOI:** 10.1155/2012/518263

**Published:** 2012-05-16

**Authors:** Sarah Dee Geiger, Charumathi Sabanayagam, Anoop Shankar

**Affiliations:** ^1^Department of Community Medicine, West Virginia University School of Medicine, Morgantown, WV 26506, USA; ^2^Singapore Eye Research Institute and Department of Clinical Sciences, Duke-NUS Graduate Medical School, Singapore 169857

## Abstract

Reduced sleep has been found to be associated with increased risk of diabetes mellitus, hypertension, cardiovascular disease (CVD), and mortality. Self-rated health (SRH) has been shown to be a predictor of CVD and mortality. However, study of the association between insufficient sleep and SRH is limited. We examined participants >18 years of age (*n* = 377, 160) from a representative, cross-sectional survey (2008 BRFSS). Self-reported insufficient sleep in the previous 30 days was categorized into six groups. The outcome was poor SRH. We calculated odds ratios ((OR) (95% confidence interval (CI)) of increasing categories of insufficient rest/sleep, taking zero days of insufficient sleep as the referent category. We found a positive association between increasing categories of insufficient sleep and poor SRH, independent of relevant covariates. In the multivariable-adjusted model, compared to 0 days insufficient sleep, the OR (95% CI) of poor SRH was 1.03 (0.97–1.10) for 1–6 days, 1.45 (1.34–1.57) for 7–13 days, 2.12 (1.97–2.27) for 14–20 days, 2.32 (2.09–2.58) for 21–29 days, and and 2.71 (2.53–2.90) for 30 days of insufficient sleep in the prior 30 days (*P*-trend <0.0001). In a nationally representative sample, increasing categories of insufficient sleep were associated with poor SRH.

## 1. Introduction

Sleep loss, long-term sleep deprivation, and insufficient rest/sleep are pervasive problems in developed countries today. Short sleep duration and insufficient rest/sleep may also be a manifestation of underlying sleep-disordered breathing (SDB). Reduced sleep has been found to be associated with increased risk of diabetes mellitus, hypertension, cardiovascular disease (CVD), and cardiovascular and all-cause mortality [1–5]. Similarly, self-rated health (SRH) has been shown to be a consistent predictor of cardiovascular disease and cardiovascular and overall mortality [6–9]. Self-rated health (SRH) is a commonly used subjective measure of health used in epidemiological studies as well as a secondary outcome in clinical trials to examine changes in subjective health status [6–10]. Epidemiological studies from several countries and social and cultural settings have shown that SRH is a valid and consistent predictor of CVD and cardiovascular and overall mortality [6–9]. Due to its predictive ability of future risk of mortality as well as objective health outcomes, an Institute of Medicine (IOM) report in 2001 recommended the inclusion of SRH measurement in national surveys in the USA as a way of tracking the subjective health status of Americans [11]. 

However, the mechanisms underlying the reported association between SRH and CVD and mortality are not known. It is possible that at least part of the association between insufficient sleep and mortality may be mediated by poor SRH—although, to our knowledge, no study has examined this topic. In this context, we examined association between insufficient rest/sleep and SRH in a large multiethnic survey of US adults after adjusting for main confounders.

## 2. Methods

### 2.1. Study Population

The Behavioral Risk Factor Surveillance System (BRFSS) is a federally funded, nationally representative survey of the civilian, noninstitutionalized, adult population aged 18 years or older. The survey is designed and conducted annually by the CDC in collaboration with the state health departments to monitor health-related behaviors and risk factors in the US population. The survey selects state-specific probability samples of households using a multistage cluster design to produce a nationally representative sample. The BRFSS uses random-digit dialing within blocks of telephone numbers to identify a probability sample of households with telephones in each state. In each household, one adult is randomly identified and interviewed. All 50 states, in addition to the District of Columbia, and the three US territories participated in the 2008 BRFSS. Detailed description of the BRFSS survey sample selection and study methodology are available online [12]. In 2008, the median cooperation rate was 75.0% and the median overall response rate was 53.3% [12]. All participants provided informed consent and this study was approved by the West Virginia University Institutional Review Board. 

 To examine the association between insufficient rest/sleep and SRH, out of the 408,068 BRFSS participants who were aged ≥18 years and not pregnant, we further excluded subjects with missing information on variables included in the multivariable model, including insufficient rest/sleep, SRH, body mass index, smoking, education, employment, physical activity, or physician-diagnosed cardiovascular disease. This resulted in 377,160 subjects with complete covariate data for the current analysis. 

### 2.2. Main Outcome of Interest: SRH

 In 2008, BRFSS included a new question on self-rated health, which asks, “Would you say that in general your health is excellent, very good, good, fair, poor, or do not know/not sure?” SRH is also termed “general health” because it refers to an individual's perception of his or her own health. The SRH continuum has been shown to be a reliable indicator of a person's objective or true health status [13]. A negative association between SRH and all-cause mortality has also been established [14].

### 2.3. Exposure Measurements

 The 2008 BRFSS survey included the question, “During the past 30 days, for about how many days have you felt you did not get enough rest or sleep?” Data from all sites were aggregated, and the numbers of days of perceived insufficient rest or sleep were categorized into six groups as zero days, 1–6 days, 7–13 days, 14–20 days, 21–29 days, and 30 days. This question was previously tested and validated in the 2006 BRFSS survey in four states (Delaware, Hawaii, New York, and Rhode Island) [15]. Also, national estimates of insufficient rest or sleep according to these categories have been recently published by the CDC [16]. 

 Age, gender, race/ethnicity, smoking status, alcohol intake, level of education, employment status, income, and physical activity were assessed using a standardized questionnaire. Individuals who had not smoked ≥100 cigarettes in their lifetimes were classified as never smokers; those who had smoked ≥100 cigarettes in their lifetimes were classified as former smokers or current smokers based on their response to the question on current smoking. Heavy alcohol drinking was defined as men who reported having more than 2 drinks per day, or more than 1 drink per day for women. Education was categorized into below high school, high school, or above high school education. Employment status was categorized as employed, unemployed, retired, unable to work, homemaker, or student. Body mass index (BMI) was categorized into <25, 25–29, ≥30 kg/m^2^. Subjects were classified as having no regular exercise if they reported not to be participating in any physical activities such as running, calisthenics, golf, gardening, or walking for exercise during the previous month. People married at the time of the survey were classified as married, while widows and divorcees were coded as unmarried. The annual household income variable was reported in quintiles as follows: less than $15,000, $15,000–$24,999, $25,000–$34,999, $35,000–$49,999, $50,000 or more. Depression (description used in BRFSS questionnaire: depression, major depression, dysthymia, or minor depression) and anxiety (description used in BRFSS questionnaire: acute stress disorder, anxiety, generalized anxiety disorder, obsessive-compulsive disorder, panic disorder, phobia, posttraumatic stress disorder, or social anxiety disorder) were categorized into dichotomous, yes/no variables. 

### 2.4. Statistical Analysis

We examined the characteristics of the study sample by calculating variable frequencies. Perceived insufficient rest/sleep was categorized into six groups as zero days, 1–6 days, 7–13 days, 14–20 days, 21–29 days, and 30 days. Our single outcome of interest was SRH. We used logistic regression models to calculate odds ratio ((OR) (95% confidence interval (CI)) of the outcome of interest associated with increasing categories of insufficient rest/sleep, taking zero days of insufficient rest/sleep as the referent category. We used two logistic regression models: the unadjusted model and the multivariable model, adjusting for sex (men, women), age (years), race-ethnicity (non-Hispanic white, non-Hispanic black, Hispanic, other), education categories (<high school, high school, >high school), (employed, unemployed, retired, unable to work, homemaker, student), income category (quintiles), marital status (no, yes), smoker (never, former, current), heavy drinker (no, yes), no regular exercise (yes, no), BMI (<25, 25–29, ≥30 kg/m^2^), CVD (no, yes), depression (no, yes), and anxiety (no, yes). Trends in the OR of the outcome across increasing insufficient rest/sleep category were determined by modeling these categories as an ordinal variable. Appropriate BRFSS survey weights that account for unequal probabilities of selection, oversampling, and nonresponse were applied for all analyses using SUDAAN (version 8.0; Research Triangle Institute, Research Triangle Park, NC) and SAS (version 9.2; SAS Institute, Cary, NC) software; SEs were estimated using the Taylor series linearization method.

## 3. Results

Table 1 presents characteristics of the study population. Nearly half of the study population were women, and about 17% were 65 years of age or older. Blacks comprised 9.7% of the study population, whereas Hispanics made up 14.4%. Over 60% of study participants had above high school education and approximately the same percentage were employed. About 57% were current smokers, 5.3% were heavy drinkers, 63.1% were overweight or obese, and 25% reported no regular exercise. Finally, 8.6% of study subjects fell into the lowest income quintile and 8.2% reported having CVD. 

**Figure 1:**
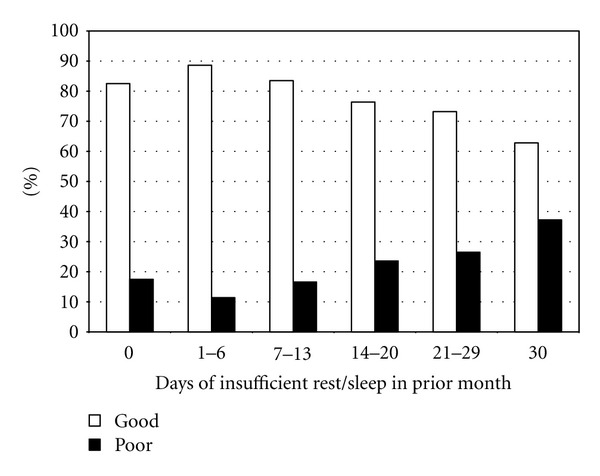
Insufficient rest/sleep by SRH. X-axis: Categories of insufficient rest or sleep in the past month. Y-axis: Weighted proportion (%) of subjects within each insufficient rest/sleep category.

Figure 1 presents the unadjusted distribution of SRH by categories of insufficient rest/sleep. Percentage of those with good SRH decreases across increasing categories of insufficient rest/sleep, whereas the opposite is true for individuals with poor SRH. 

Table 2 presents the association between increasing categories of insufficient rest/sleep and poor SRH. We found that in the unadjusted model, a positive association between increasing categories of insufficient rest/sleep and poor SRH was evident only from ≥14 days of insufficient rest/sleep. However, with multivariable adjustment, a linear dose-response association was evident (*P*-trend <0.0001). 

Tables 3, 4, 5, and 6 present the association between insufficient rest/sleep and poor SRH within stratified subgroups of gender, age, BMI, and race-ethnicity categories. We found that the positive association between insufficient rest/sleep and poor SRH persisted across all these stratified subgroups. Also, there was no statistically significant interaction between insufficient rest/sleep and SRH by age group, gender, or race/ethnciity (all *P*-interactions >0.10).

## 4. Discussion

 In a multiethnic, representative sample of US adults, increasing categories of insufficient rest/sleep were shown to be positively associated with poor SRH overall and the association persisted when stratified by (1) gender, (2) age, (3) BMI, and (4) race-ethnicity. The positive association was present even when adjusting for confounding factors such as sex, age, race-ethnicity, education, employment status, income level, marital status, smoking, heavy drinking, exercise, BMI, depression, and anxiety. Our results indirectly suggest that poor SRH may be a mediator of the association between insufficient sleep and CVD and mortality [6, 7, 9, 14]. However, this hypothesis needs to be directly tested in a future study that simultaneously measures insufficient rest/sleep, SRH, as well as CVD and mortality outcomes. 

 Internal validity for the detected association between perceived insufficient rest/sleep and poor SRH is high due to the magnitude of association, its independence from potential confounders such as sex, age, race-ethnicity, smoking, drinking, BMI, depression, and others. The presence of a positive dose-response trend and the persistence of the association in stratified analyses by subgroups of gender, age, BMI, and race-ethnicity suggest that our findings are not due to chance. 

 Only a few studies have examined the association between sleep and SRH and their results have been inconsistent. Segovia et al., in a study of 3,300 subjects from St. John's metropolitan area in Canada, found an association between sleep duration of less than 7 hours or greater than 8 hours and poor SRH [17]. However, the authors did not adjust for possible confounders such as ethnicity or depressive symptoms. Steptoe et al., in a study of more than 17,000 university students, found an association between only short sleep duration and poor SRH [18]. A study by Jean-Louis et al. found no association between sleep duration and poor SRH, but this study used a sample of only 273 California residents [19]. The current study found a significant association between insufficient sleep and poor SRH using a sample size of 377,160, which is the largest study on this topic to date. Also, due to our large sample size, we were able to conduct detailed subgroup analyses by gender, age, race-ethnicity, and BMI and confirm that the sleep-SRH association was present consistently within these subgroups. 

Studies have reported that poor SRH is an independent predictor of mortality [9, 14]. Studies have also shown that insufficient sleep predicts mortality [19]. Based on our findings, a corollary observation is that at least part of the association between poor SRH and mortality may be mediated by insufficient sleep. Also, insufficient rest/sleep may be related to sleep deficiency, which has been recently shown by Sorensen et al. to be related to multiple health outcomes as well as poor SRH [20]. The sleep deficiency/sleep adequacy constructs are similar to insufficient sleep but have historically been assessed using the Medical Outcomes Study sleep scale [21]. There is a need for future studies to examine the relation between similar measures of insufficient sleep and sleep adequacy and how they may jointly influence multiple health and well-being outcomes. 

 Strengths of the study include its large sample size, standardized data collection methods, and the availability of data on potentially confounding conditions such as BMI, depression, and anxiety. Also, the sample is nationally representative, with study samples from all 50 US states, the District of Columbia, and the three territories, men and women, and all race-ethnicities, which increases the study's generalizability. The first limitation of the study is its cross-sectional nature. Therefore, assertions about temporality or causation such as a sleep duration recommendations cannot be established here. Second, all BRFSS data are self-reported, which may lead to misclassification and therefore biased estimates. Third, the 2008 BRFSS did not include items on potential confounders such as shift work, sleep disorders, or relevant medication usage, which therefore were not controlled in this analysis. Fourth, the insufficient rest/sleep variable introduces a limitation due to subjectivity. Respondents may conceptualize the words “insufficient” and “rest” differently. Some respondents may have conceptualized true sleep when self-reporting, while others may have considered sleep plus rest, such as time spent watching television. The subjectivity makes comparison to studies of objective measures of insufficient sleep, including polysomnography, difficult. Fifth, the global SRH measure is also subjective and nonspecific, characteristics that may be viewed as limitations. However, the SRH measure's reliability and repeatability has been confirmed in multiple studies across several continents and various cultural settings [13, 22]. Furthermore, SRH has the potential to capture dimensions of health that are not covered in traditional, objective clinical examinations, which are more likely to be focussed around a particular organ system (e.g., cardiovascular system or gastrointestinal system), and may also account for slowly progressing diseases that evade clinical diagnoses [23]. Sixth, the BRFSS survey did not collect information on habitual sleep duration. Therefore, unfortunately, we are not able to examine if the relationship between insufficient sleep and poor SRH is modified by habitual sleep duration. Finally, the insufficient rest/sleep item newly included in the 2008 BRFSS has not been widely used or tested and therefore its reliability and validity remains questionable. However, widely tested and validated sleep quality instruments such as the Pittsburgh Sleep Quality Index and the Epworth Sleepiness Scale do include similar items on perceived rest/sleep. Future studies examining the association between the insufficient rest/sleep BRFSS item and objectively measured sleep duration (e.g., actigraphy) are needed. Future research should also include longitudinal studies to establish causation potentially including sleep duration interventions [10]. 

 In summary, in a multiethnic, nationally representative sample of US adults, increasing categories of perceived insufficient rest/sleep was found to be positively associated with poor SRH. This association persisted independent of sex, age, race-ethnicity, education, employment, income, marital status, smoking, heavy alcohol intake, lack of exercise, overweight/obese BMI, CVD, depression, and anxiety. Based on this study, self-reported insufficient rest/sleep appears to be a strong predictor of SRH.

## Figures and Tables

**Table 1:** Characteristics of the study sample (*n* = 377, 160).

Characteristic	Percentages ± standard error (SE)
Women	49.6 ± .2
65 and over	16.7 ± .1
Race/Ethnicity	
Non-Hispanic white	68.5 ± .2
Non-Hispanic black	9.7 ± .1
Hispanic	14.4 ± .2
Other	6.8 ± .1

Education level	
Below high school	10.8 ± .1
High school	28.6 ± .2
Above high school	60.6 ± .2

Employment status	
Employed	60.8 ± .2
Unemployed	6.0 ± .1
Retired	15.8 ± .1
Unable to work	5.0 ± .1
Homemaker	7.6 ± .1
Student	4.8 ± .1

Annual household income	
<$15,000	8.6 ± .1
$15,000–$24,999	13.7 ± .1
$25,000–$34,999	9.9 ± .1
$35,000–$49,999	13.0 ± .1
≥$50,000	44.3 ± .2
Married	60.2 ± .2

Smoking	
Never smoker	18.7 ± .1
Former smoker	24.6 ± .1
Current smoker	56.8 ± .2
Heavy drinker	5.3 ± .1
No regular exercise	24.9 ± .1

Body mass index categories	
Normal	36.9 ± .2
Overweight or Obese	63.1 ± .2
CVD	8.2 ± .1
Depression	1.4 ± .03
Anxiety	1.1 ± .03

Insufficient rest/sleep	
0 days	30.5 ± .2
1–6 days	29.7 ± .2
7–13 days	11.9 ± .1
14–20 days	13.4 ± .1
21–29 days	3.5 ± .1
30 days	11.0 ± .1

Self-rated health	
Excellent	20.8 ± .1
Very good	33.1 ± .2
Good	30.1 ± .2
Fair	11.7 ± .1
Poor	4.2 ± .1

**Table 2:** Association between insufficient rest/sleep and poor SRH.

Categories of insufficient rest/sleep	No. at risk	No. with poor SRH	Unadjusted odds ratio (95% confidence intervals)	Multivariable odds ratio (95% confidence intervals)*
Whole cohort	377160	70778		
0 days	134596	23570	1 (referent)	1 (referent)
1–6 days	106560	12190	0.64 (0.61–.68)	1.03 (0.97–1.10)
7–13 days	39751	6580	0.89 (0.83–.95)	1.45 (1.34–1.57)
14–20 days	44781	10580	1.35 (1.28–1.43)	2.12 (1.97–2.27)
21–29 days	12152	3224	1.54 (1.41–1.67)	2.32 (2.09–2.58)
30 days	39320	14634	2.52 (2.39–2.65)	2.71 (2.53–2.90)

*P*-trend			<0.0001	<0.0001

*Adjusted for sex (men, women), age (years), race-ethnicity (non-Hispanic white, non-Hispanic black, Hispanic, other), education categories (<high school, high school, >high school), employment status (employed, unemployed, retired, unable to work, homemaker, student), income category (quintiles), marital status (no, yes), smoker (never, former, current), heavy drinker (no, yes), no regular exercise (yes, no), body mass index categories (<25, 25–29, ≥30 kg/m^2^), CVD (no, yes), depression (no, yes), and anxiety (no, yes).

**Table 3:** Association between insufficient rest/sleep and poor SRH, by gender.

Categories of insufficient rest/sleep	No. at risk	No. with poor SRH	Unadjusted odds ratio (95% confidence intervals)	Multivariable odds ratio (95% confidence intervals)*
Men	147300	26309		
0 days	57456	9970	1 (referent)	1 (referent)
1–6 days	41088	4386	0.63 (.58–.68)	1.06 (.97–1.17)
7–13 days	14614	2175	0.82 (.74–.91)	1.41 (1.24–1.61)
14–20 days	15989	3557	1.23 (1.13–1.35)	2.02 (1.81–2.25)
21–29 days	4155	1011	1.50 (1.30–1.73)	2.42 (2.03–2.90)
30 days	13988	5210	2.53 (2.33–2.75)	2.70 (2.42–3.00)

*P*-trend			<0.0001	<0.0001

Women	229860	44469		
0 days	77140	13600	1 (referent)	1 (referent)
1–6 days	65472	7804	0.66 (.62–.70)	1.01 (.94–1.09)
7–13 days	25137	4405	0.94 (.87–1.02)	1.50 (1.37–1.65)
14–20 days	28.792	7023	1.45 (1.35–1.55)	2.21 (2.03–2.42)
21–29 days	7997	2213	1.55 (1.40–1.71)	2.26 (2.01–2.54)
30 days	25322	9424	2.48 (2.33–2.65)	2.72 (2.50–2.96)

*P*-trend			<0.0001	<0.0001

*Adjusted for sex (men, women), age (years), race-ethnicity (non-Hispanic white, non-Hispanic black, Hispanic, other), education categories (<high school, high school, >high school), (employed, unemployed, retired, unable to work, homemaker, student), income category (quintiles), marital status (no, yes), smoker (never, former, current), heavy drinker (no, yes), no regular exercise (yes, no), body mass index categories (<25, 25–29, ≥30 kg/m2), CVD (no, yes), depression (no, yes), and anxiety (no, yes).

**Table 4:** Association between insufficient rest/sleep and poor SRH, by age category.

Categories of insufficient rest/sleep	No. at risk	No. with poor SRH	Unadjusted odds ratio (95% confidence intervals)	Multivariable odds ratio (95% confidence intervals)*
<65 years of age	265278	40975		
0 days	70920	8961	1 (referent)	1 (referent)
1–6 days	81902	7186	0.74 (.69–.79)	1.03 (.95–1.12)
7–13 days	32992	4416	1.04 (.96–1.13)	1.42 (1.29–1.57)
14–20 days	37806	7648	1.64 (1.53–1.76)	2.09 (1.91–2.27)
21–29 days	10431	2460	1.92 (1.74–2.13)	2.34 (2.07–2.64)
30 days	31227	10304	3.04 (2.84–3.25)	2.66 (2.44–2.89)

*P*-trend			<0.0001	<0.0001

≥65 years of age	111882	29803		
0 days	63676	14609	1 (referent)	1 (referent)
1–6 days	24658	5004	0.90 (.84–.96)	1.06 (.98–1.14)
7–13 days	6759	2164	1.61(1.46–1.78)	1.61 (1.44–1.80)
14–20 days	6975	2932	2.45 (2.23–2.69)	2.28 (2.07–2.52)
21–29 days	1721	764	2.48 (2.07–2.98)	2.19 (1.79–2.69)
30 days	8093	4330	3.85 (3.54–4.19)	2.96 (2.68–3.26)

*P*-trend			<0.0001	<0.0001

*Adjusted for sex (men, women), age (years), race-ethnicity (non-Hispanic white, non-Hispanic black, Hispanic, other), education categories (<high school, high school, >high school), (employed, unemployed, retired, unable to work, homemaker, student), income category (quintiles), marital status, smoker (never, former, current), heavy drinker (no, yes), no regular exercise (yes, no), body mass index categories (<25, 25–29, ≥30 kg/m^2^), CVD (no, yes), depression (no, yes), and anxiety (no, yes).

**Table 5:** Association between insufficient rest/sleep and poor SRH, by BMI.

Categories of insufficient rest/sleep	No. at risk	No. with poor SRH	Unadjusted odds ratio (95% confidence intervals)	Multivariable odds ratio (95% confidence intervals)*
Normal weight (BMI <25 kg/m^2^)	134529	19836		
0 days	49373	7383	1 (referent)	1 (referent)
1–6 days	39532	3346	0.53 (.48–.58)	0.92 (.82–1.02)
7–13 days	14100	1779	0.82 (.73–.92)	1.52 (1.31–1.76)
14–20 days	15100	2697	1.12 (1.01–1.25)	2.03 (1.78–2.33)
21–29 days	4033	822	1.32 (1.13–1.55)	2.27 (1.88–2.73)
30 days	12391	3809	2.40 (2.18–2.66)	2.85 (2.50–3.24)

*P*-trend			<0.0001	<0.0001

Overweight/Obese (BMI >25 kg/m^2^)	242631	50942		
0 days	85223	16187	1 (referent)	1 (referent)
1–6 days	67028	8844	0.70 (.66–.75)	1.09 (1.01–1.17)
7–13 days	25651	4801	0.92 (.85–.99)	1.43 (1.31–1.57)
14–20 days	29681	7883	1.45 (1.35–1.54)	2.15 (1.98–2.33)
21–29 days	8119	2402	1.60 (1.44–1.77)	2.35 (2.07–2.66)
30 days	26929	10825	2.53 (2.38–2.68)	2.66 (2.46–2.87)

*P*-trend			<0.0001	<0.0001

*Adjusted for sex (men, women), age (years), race-ethnicity (non-Hispanic white, non-Hispanic black, Hispanic, other), education categories (<high school, high school, >high school), (employed, unemployed, retired, unable to work, homemaker, student), income category (quintiles), marital status, smoker (never, former, current), heavy drinker (no, yes), no regular exercise (yes, no), body mass index categories (<25, 25–29, ≥30 kg/m^2^), CVD (no, yes), depression (no, yes), and anxiety (no, yes).

**Table 6:** Association between insufficient rest/sleep and poor SRH, by race-ethnicity.

Categories of insufficient rest/sleep	No. at risk	No. with poor SRH	Unadjusted odds ratio (95% confidence intervals)	Multivariable odds ratio (95% confidence intervals)*
Non-Hispanic white	299905	50401		
0 days	106395	17161	1 (referent)	1 (referent)
1–6 days	86387	8197	0.51 (0.48–.53)	0.89 (0.84–.95)
7–13 days	32055	4565	0.76 (0.71–.81)	1.35 (1.25–1.46)
14–20 days	35899	7756	1.29 (1.22–1.37)	2.10 (1.95–2.26)
21–29 days	9884	2405	1.55 (1.42–1.70)	2.39 (2.13–2.69)
30 days	29285	10317	2.59 (2.45–2.74)	2.85 (2.64–3.06)

*P*-trend			<0.0001	<0.0001

Non-Hispanic black	28966	7727		
0 days	9916	2312	1 (referent)	1 (referent)
1–6 days	7822	1547	0.83 (.72–.96)	1.27 (1.06–1.51)
7–13 days	3090	800	1.09 (.90–1.32)	1.56 (1.25–1.95)
14–20 days	3357	1123	1.67 (1.40–2.00)	2.53 (2.04–3.14)
21–29 days	889	324	1.75 (1.29–2.39)	2.88 (2.08–3.99)
30 days	3892	1621	2.39 (2.05–2.79)	2.75 (2.28–3.32)

*P*-trend			<0.0001	<0.0001

Hispanic	24810	7542		
0 days	9707	2547	1 (referent)	1 (referent)
1–6 days	6361	1560	1.10 (.95–1.27)	1.42 (1.20–1.68)
7–13 days	2305	739	1.38 (1.12–1.69)	1.82 (1.45–2.28)
14–20 days	2723	978	1.57 (1.31–1.88)	2.21 (1.80–2.72)
21–29 days	612	230	1.39 (1.01–1.91)	1.78 (1.25–2.55)
30 days	3102	1488	2.51 (2.12–2.98)	2.54 (2.07–3.12)

*P*-trend			<0.0001	<0.0001

Other	20556	4361		
0 days	7280	1268	1 (referent)	1 (referent)
1–6 days	5346	763	0.62 (.49–.77)	0.89 (.69–1.13)
7–13 days	2051	408	1.17 (.86–1.59)	1.69 (1.19–2.39)
14–20 days	2515	631	1.53 (1.21–1.93)	1.85 (1.39–2.47)
21–29 days	664	236	2.19 (1.56–3.06)	2.55 (1.72–3.77)
30 days	2700	1055	2.66 (2.13–3.33)	2.42 (1.84–3.19)

*P*-trend			<0.0001	<0.0001

*Adjusted for sex (men, women), age (years), race-ethnicity (non-Hispanic white, non-Hispanic black, Hispanic, other), education categories (<high school, high school, >high school), (employed, unemployed, retired, unable to work, homemaker, student), income category (quintiles), marital status, smoker (never, former, current), heavy drinker (no, yes), no regular exercise (yes, no), body mass index categories (<25, 25–29, ≥30 kg/m^2^), CVD (no, yes), depression (no, yes), and anxiety (no, yes).
